# Composite restorations placed in non‐carious cervical lesions—Which cavity preparation is clinically reliable?

**DOI:** 10.1002/cre2.310

**Published:** 2020-09-13

**Authors:** Anne‐Katrin Lührs, Silke Jacker‐Guhr, Hüsamettin Günay, Peggy Herrmann

**Affiliations:** ^1^ Department of Conservative Dentistry, Periodontology and Preventive Dentistry Hannover Medical School Hannover Germany

**Keywords:** cavity preparation design, class V‐lesions, in‐vivo study, NCCL, non‐carious cervical lesions, USPHS criteria

## Abstract

The purpose of this in‐vivo study was to evaluate the clinical performance of restorations placed in non‐carious cervical lesions (NCCLs), using different cavity preparation designs, after 7.7 years. A total of 85 NCCLs with coronal margins in enamel and cervical margins in dentin were randomly assigned to the following treatment protocols: dentin surface cleaning, dentin surface roughening with round bur plus flowable composite, dentin surface roughening/cervical groove preparation with round bur, dentin surface roughening/cervical groove preparation with round bur plus flowable composite. After enamel beveling and selective enamel etching, the defects were restored with composite. The restorations were assessed by two independent, calibrated and blinded investigators, using modified USPHS criteria. At 7 years (7.7 (± 0.35)), a total of 64 restorations (75.3%) were available for follow‐up examination. The total retention rate, irrespective of the test groups, was 82.8%. Restorations placed without any preparation showed the highest loss rate (27.8%). Esthetic appearance, marginal adaptation, anatomic form and marginal discoloration did not differ significantly between the groups. Composites are long‐term stable materials for restoring NCCLs. Restorations placed without any dentin preparation (cavity cleaning only) showed the highest loss rate.

## 1 INTRODUCTION

Non‐carious cervical lesions (NCCLs) occur in all age groups (Borcic, Anic, Urek, & Ferreri, 2004; Kolak et al., 2018; Yang et al., 2016), but epidemiological studies have shown an increase of these defects at an advanced age (Kolak et al., 2018; Yang et al., 2016). In the group of over 55‐year‐olds, 94.7% of all patients examined have NCCLs, and one‐third of them even show more than three lesions (Kolak et al., 2018).

The development of NCCLs is a multifactorial process. The most common causes are erosion, abrasion and abfraction due to occlusal interferences (Osborne‐Smith, Burke, & Wilson, 1999). NCCLs are often associated with hypersensitivities, esthetic impairments, and the progression of cervical tooth structure loss. Especially when the causes of such lesions include erosion, a change in the toothbrushing technique alone will usually not prevent the existing defect from progressing (Perez et al., 2012). Brushing frequency, contact pressure, toothpaste abrasiveness and toothbrush hardness are cofactors for the development of NCCLs (Dickson, Vandewalle, Lien, Dixon, & Summitt, 2015; Sadaf & Ahmad, 2014; Wiegand & Schlueter, 2014). NCCLs are frequently located on vestibular surfaces of premolars, followed by canines. Upper first premolars show the highest prevalence, while second molars and anterior teeth are least often affected (Aw, Lepe, Johnson, & Mancl, 2002; Borcic et al., 2004; Igarashi, Yoshida, & Kanazawa, 2017; Kolak et al., 2018). When restorative treatment of such defects is indicated, various factors may negatively influence the long‐term stability of the adhesive restorations placed.

Cervical defects show structural differences from normal dentin, resulting from exposure to the oral environment (Palamara, Palamara, Messer, & Tyas, 2006; Walter et al., 2014). A heterogeneous, hypermineralized surface is caused by prolonged exposure of dentin to saliva (El‐din, Miller, & Griggs, 2004). It is characterized by high phosphate and low carbonate contents, a high proportion of crystalline structures and partially denatured dentin (Karan, Yao, Xu, & Wang, 2009). Due to dentin sclerosis, the bond strength of adhesive composite restorations to dentin may be lower, which in turn might lead to a higher restoration loss rate (Aw et al., 2002).

Furthermore, flexural forces occur in cervical cavities with incisal margins in enamel and cervical margins in dentin, as a result of the different moduli of elasticity of these two structures (enamel: 84.1 GPa, dentin: 16.6–18.6 GPa), so that the restorative material used has to meet these special requirements (Craig & Peyton, 1958; Fennis et al., 2005).

Basically, glass ionomer cements, compomers and composites in various viscosities can be used to restore NCCLs (Cieplik et al., 2017). However, composites are the materials of choice, due to their esthetic and physical properties (Pecie, Krejci, Garcia‐Godoy, & Bortolotto, 2011; Perez et al., 2012). In addition to the abovementioned factors, the cavity preparation design (Hakimeh, Vaidyanathan, Houpt, Vaidyanathan, & von Hagen, 2000), adhesive system and layering technique used influence the long‐term stability of such composite restorations (Borges, Borges, Xavier, Bottino, & Platt, 2014; Boushell et al., 2016; Correia et al., 2018). Since the development of NCCLs is usually a multifactorial process involving different substrates, i.e., enamel and dentin, a combination of high‐ and low‐viscosity composites and the use of an incremental technique are considered to be the optimal treatment (Mullejans, Lang, Schuler, Baldawi, & Raab, 2003; Perez, 2010). Cavity preparation design is another influencing factor: U‐shaped cavities show less microleakage than V‐shaped cavities in vitro (Hakimeh et al., 2000); this is in part attributable to the fact that composite is more effectively packed when there are parallel cavity walls.

To date, only few in‐vivo data on Class V restorations involving composites of different viscosities or comparisons of different preparation designs have been published (Cieplik et al., 2017; Correia et al., 2018; Karaman, Yazici, Ozgunaltay, & Dayangac, 2012; Li, Jepsen, Albers, & Eberhard, 2006; Mullejans et al., 2003; Szesz, Parreiras, Martini, Reis, & Loguercio, 2017). Therefore, the aim of this prospective randomized clinical study was to investigate the influence of dentin surface pretreatment (cleaning vs. roughening vs. groove preparation) and the application of a flowable composite on the clinical long‐term stability of cervical restorations.

The null hypothesis which was set forth was that the different pretreatment modes do not influence the retention rate and the clinical behavior (based on modified USPHS criteria) of composite restorations placed in NCCLs.

## 2 MATERIALS AND METHODS

All procedures performed in this in‐vivo study were in accordance with the ethical standards of the institutional research committee (No.: 4613) of the Hannover Medical School. All participants gave their written informed consent before treatment.

This prospective randomized clinical study focused on follow‐up examinations of composite restorations placed in NCCLs after 7.7 years of intraoral retention. Twenty‐four patients with a total of 85 NCCLs requiring treatment participated in the study. The clinical selection of the cervical defects was based on lesion depth. Following the Tooth Wear Index by Smith and Knight (1984), cervical defects of at least 1 mm in depth were included in the study. Table 1 summarizes all inclusion and exclusion criteria. The patient recruitment and all treatments took place between September 2007 and May 2008. The last follow‐up examination was carried out in June 2015.

**TABLE 1:** Inclusion and exclusion criteria

Inclusion criteria	Exclusion criteria
Lesion depth ≥ 1 mm	Lack of written informed consent to participate
Cervical hypersensitivities	Underage patients
Restoration of tooth contour to prevent periodontal damage	Carious cervical lesions
Esthetic reasons	Pregnant or nursing women
Lesions with coronal margins in enamel and cervical margins in dentin	Allergies to components of the materials used
	Infectious diseases
	Mucosal diseases with unclear diagnosis
	Inadequate oral hygiene
	Bruxism
	High caries activity
	Non‐vital pulp
	Severe periodontal diseases
	Severe dysgnathia/traumatic occlusion
	No antagonist/adjacent tooth present
	Undergoing orthodontic treatments
	Undergoing bleaching procedures

At baseline, risk factors for extrinsic discolorations were documented, the teeth were tested for vitality, and the initial clinical situation was photographed. All patients received oral hygiene instructions prior to restorative treatment.

The cavities included had coronal margins in enamel and cervical margins in dentin and were randomly assigned (randomization list) to one of the four test groups. The groups differed in the treatment protocols applied before composite application. The different groups are displayed in Table 2.

**TABLE 2:** Overview over the four different treatment groups, which were applied before the high viscous composite was placed

Group	Code	
1	CLEAN	Dentin surface cleaning with fluoride‐free prophylaxis paste, application of adhesive system after selective enamel etching with phosphoric acid
2	PREP_FLOW	Dentin surface roughening with round bur, application of adhesive system after selective enamel etching with phosphoric acid, application of a thin layer flowable composite to the cervical area
3	GROOVE	Dentin surface roughening/cervical groove preparation with round bur, application of adhesive system after selective enamel etching with phosphoric acid
4	GROOVE_FLOW	Dentin surface roughening/cervical groove preparation with round bur, application of adhesive system after selective enamel etching with phosphoric acid, application of a thin layer flowable composite to the cervical groove

**FIGURE 1:**
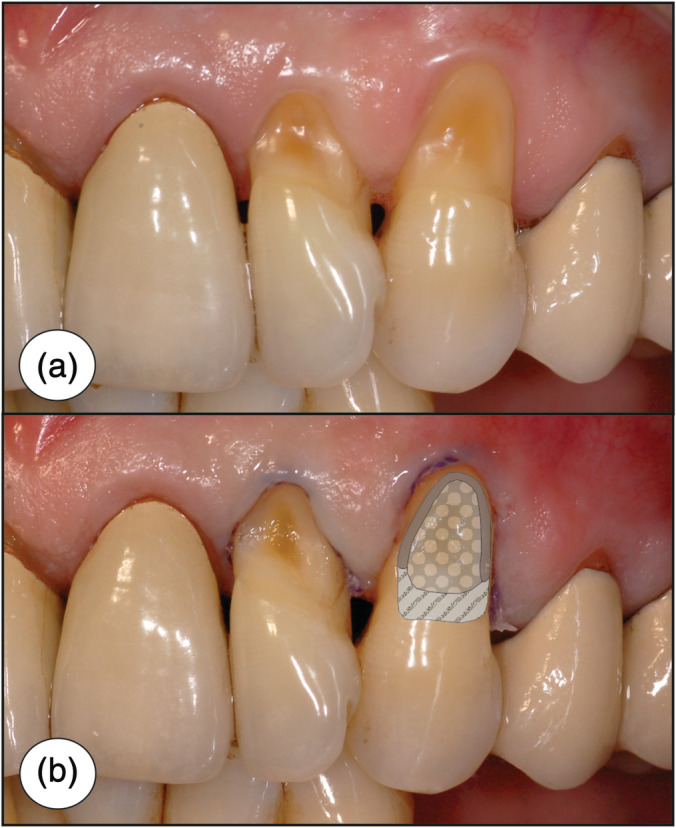
(a) Clinical situation before treatment, NCCLs located at teeth 22 and 23, no gingival inflammation present. (b) Clinical situation after surface roughening/groove preparation with retraction cord in place. Cavity preparation design is illustrated at tooth 23. Grey = small cervical groove (groups 3 and 4 only, depth max. 0.5 mm), dotted area: roughened dentin (groups 2,3 and 4; in group 1, this area was cleaned only), striped area: beveled enamel (all groups)

Prior to restoration, all teeth were cleaned mechanically (Curette Gracey Micro #7/8 Gr #9 MF, Everedge 2.0‐SMS7/89E2, Hu‐Friedy, Tuttlingen, Germany) and with fluoride‐free prophylaxis paste (HAWE Cleanic, Kerr GmbH, Biberach, Germany) to remove organic and inorganic deposits from the surfaces. After thorough rinsing of the cavities with water for 30 s and subsequent drying, the tooth shades were determined with the dedicated shade guide of the composite used (Tetric EvoCeram, Ivoclar Vivadent, Schaan, Liechtenstein). In group 1, the dentin surfaces were not prepared; in groups 2 to 4, the surface was roughened carefully with a round carbide bur size 14/16, depending on the cavity size (H1SEM.204.014 VPE 5/ H1SEM.204.016 VPE 5, Komet Dental, Lemgo, Germany). For the preparation of the fine cervical groove in groups 3 and 4 (Figure 1), a size 010 round carbide bur (H1SEM.205.010 VPE 5, Komet Dental, Lemgo, Germany) was used. The preparation was done with low speed (2,000 rpm) without watercooling and with low pressure.

The coronal enamel margins of all cavities were bevelled approx. 1–1.5 mm, using a diamond finishing instrument (Flame No. 8862, average grain size 30 μm, Komet Dental, Lemgo, Germany) with water cooling (see Figure 1). A dry retraction cord without any hemostatic agent (Ultrapak CleanCut Size 0, Ultradent Products, Köln, Germany) was used to slightly displace the gingiva and make intrasulcular or slightly subgingival preparation margins accessible. For an atraumatic application of the retraction cord, a small packer (Fischer's Ultrapak Packer, Small 45° Packer, Ultradent Products, Köln, Germany) was used. Then, the enamel was selectively etched for 30 s with 36% phosphoric acid gel (DeTrey Conditioner 36, Dentsply DeTrey, Konstanz, Germany) and rinsed with water spray for 30 s. Dentin etching with phosphoric acid is an optional working step when using Syntac because of the self‐etching primer, which contains maleic acid (see Table 3). Therefore, dentin was pretreated with the self‐etching primer and not etched with phosphoric acid.

**TABLE 3:** Adhesive system and composite materials with Batch No., manufacturer's instructions and composition

Material	Batch no.	Manufacturer's instructions/application	Composition	Manufacturer
HAWE Cleanic without fluoride	N.N.	Application with prophylaxis cup to remove organic and inorganic deposits from the surfaces, thorough rinsing with water for 30 s, drying	Silicates, humenctant (glycerine), binder, flavour	Kerr GmbH, Biberach, Germany
DeTrey conditioner 36	1,006,002,311	Apply etching gel selectively on enamel, etching for 30 s, rinsing for 30 s, drying with light air‐flow.	Phorphoric acid, water, silicon dioxide, water	Dentsply DeTrey, Konstanz, Germany
Syntac Primer	K36299, L05849	Apply Primer to the cavity and gently rub it in. Contact time at least 15 s. Disperse excess and thoroughly dry.	Triethylene glycol dimethacrylate, polyethylene glycol dimethacrylate, maleic acid and acetone in an aqueous solution	Ivoclar Vivadent, Schaan, Liechtenstein
Syntac Adhesive	K36300, L02854	Apply Adhesive, leave it for 10 s, and thoroughly dry the cavity with an air syringe.	Polyethylene glycol dimethacrylate and glutaraldehyde in an aqueous solution	Ivoclar Vivadent, Schaan, Liechtenstein
Heliobond	K37826, L05313	Apply Heliobond and blow it to a thin layer. Light‐cure for 10 s at a minimum of 500 mW/cm^2^	Bis‐GMA, triethylene glycol dimethacrylate, stabilizers and catalysts	Ivoclar Vivadent, Schaan, Liechtenstein
Tetric EvoFlow	J22244, K03621	Maximum layer thickness: 2 mm (or 1.5 mm for Dentin shades). Light‐cure for 20 s at ≥500 mW/cm^2^ or for 10 s at ≥1,000 mW/cm^2^. Layer thickness was modified due to the study protocol applied: max. thickness 0.5 mm	Bis‐GMA, urethane dimethacrylate, decanediol dimethacrylate, barium glass, ytterbium trifluoride, mixed oxide, highly dispersed silicon dioxide, silanized, pre‐polymer, additives, catalysts, stabilizers and pigments	Ivoclar Vivadent, Schaan, Liechtenstein
Tetric EvoCeram	H13349, J27436, K00012	Maximum layer thickness: 2 mm (or 1.5 mm for Dentin shades). Light‐cure for 20 s at ≥500 mW/cm^2^ or for 10 s at ≥1,000 mW/cm^2^.	Bis‐GMA, urethane dimethacrylate, ethoxylated bisphenol‐A dimethacrylate, barium glass, ytterbium trifluoride, mixed oxide, copolymer, additives, catalysts, stabilizers and pigments, particle size approx. 0.6 μm	Ivoclar Vivadent, Schaan, Liechtenstein

After rinsing the etchant off and drying the cavity surface, the adhesive system (Syntac: Syntac Primer, Syntac Adhesive, Heliobond, Ivoclar Vivadent, Schaan, Liechtenstein) was applied and light‐cured following the manufacturer's instructions (see Table 3). In Groups 2 and 4, a thin layer of flowable composite (max. layer thickness: 0.5 mm, Tetric EvoFlow, Ivoclar Vivadent, Schaan, Liechtenstein) was applied to the cervical area/groove and light‐cured for 20 s at 1,200 mW/cm^2^ with an LED curing light (Bluephase, Ivoclar Vivadent, Schaan, Liechtenstein). Then the defects were restored with a high‐viscosity composite which includes glass microfillers with a mean particle size of 0.6 μm (Tetric EvoCeram, Ivoclar Vivadent) using an incremental technique with a maximum increment thickness of 2 mm. Each increment was light‐cured for 20 s with the above‐mentioned curing light. Table 3 shows the materials used in the study.

The restorations were finished and polished with diamond finishing burs (Flame No. 8862, average grain size 30 μm, Komet Dental, Lemgo, Germany), polishing disks (Sof‐Lex XT, 3 M Deutschland, Neuss, Germany), the EVA System (KaVo, Biberach, Germany) with oscillating files (Proxoshape PS2, Intensiv, Montagnola, Switzerland), and silicone polishers (OptraPol, Ivoclar Vivadent, Schaan, Liechtenstein).

After an average period of 7.7 years, the restorations were clinically examined by two calibrated, independent and blinded investigators, using modified USPHS criteria based on Cvar and Ryge (Table 4) (Cvar & Ryge, 2005). Also, bleeding on probing (BOP, periodontal probe GY12, Deppeler SA, Rolle, Switzerland) at six sites (mv, v, dv, mp/ml, p/l, dp/dl) around the respective tooth tested was evaluated and documented.

**TABLE 4:** Modified USPHS criteria based on Cvar and Ryge (2005)

	Esthetic appearance	Marginal adaptation	Anatomic form	Marginal discoloration	Axial contour	Secondary caries	Gingival response	Hypersensitivity
A	Good shade match between restoration and adjacent tooth structure	No marginal gap visible or detectable with an explorer	Continuous transition between restoration and tooth structure	No marginal discoloration	Restoration matches tooth shape	No signs of caries	No response	No hypersensitivity
B	Slight to moderate mismatch in shade between restoration and tooth structure, but still esthetically acceptable	Marginal gap visible, but not extending to dentin or cavity base	Discontinuous transition, dentin or cavity base not exposed	Slight partial marginal discoloration	Restoration slightly over‐ or under‐contoured	Secondary caries	Gingival response without clinical inflammation	Hypersensitivity attributable to hyperemia
C	Severe discoloration	Explorer insertable in gap, dentin or cavity base exposed	Dentin or cavity base exposed	Severe circular marginal discoloration	Restoration moderately over‐ or under‐contoured		Bleeding on probing	Pulpitis/necrosis
D		Restoration fractured, mobile, partially or totally missing			Insufficient restoration, severely over‐ or under‐contoured		Bleeding on probing and hyperplasia	

The data was statistically analyzed using the chi‐square test (*p* < 0.05, SPSS 23.0, IBM Deutschland GmbH, Ehningen, Germany). The inter‐rater reliability was tested with Cohen's kappa coefficient.

## 3 RESULTS

**FIGURE 2:**
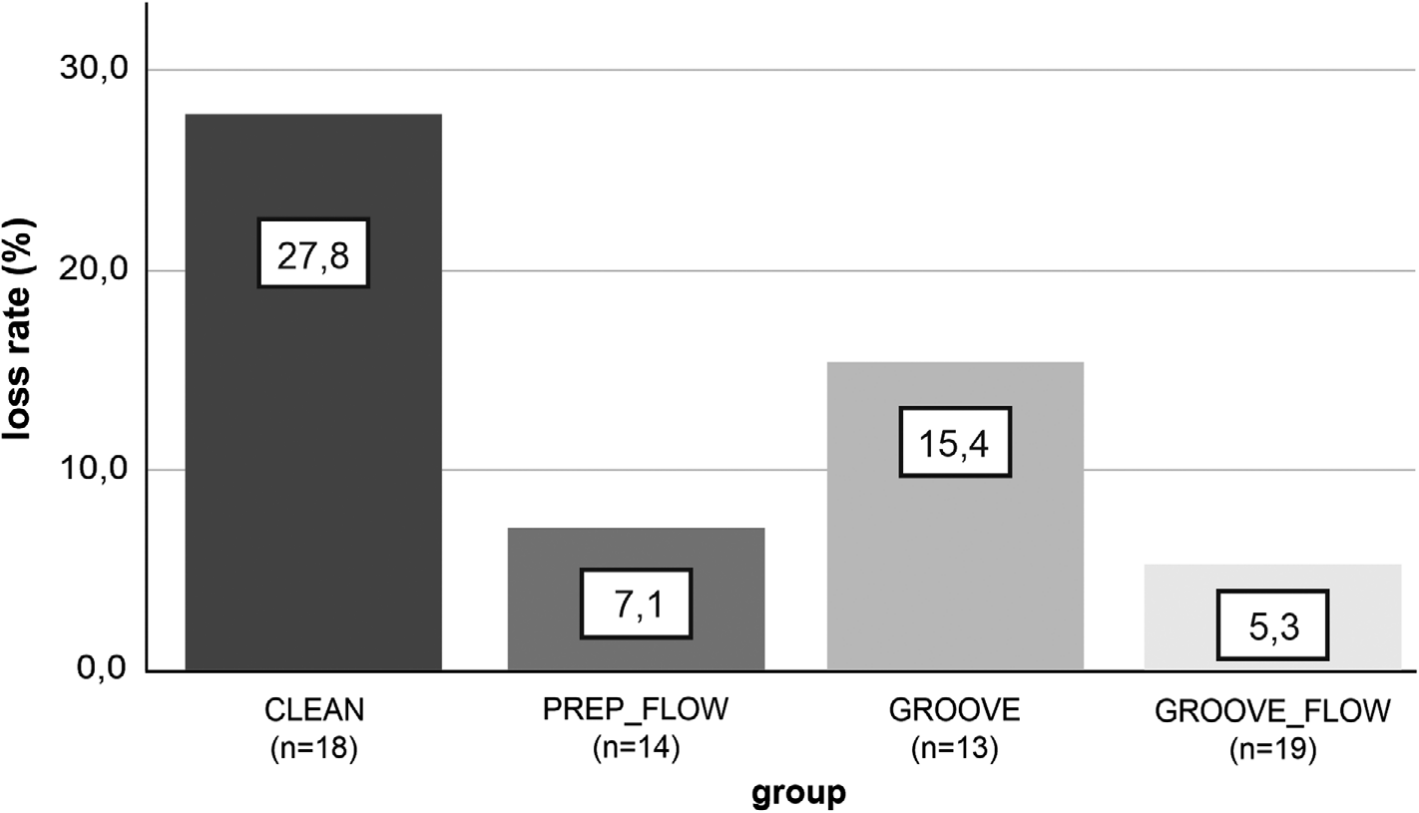
Comparison of the loss rates (in %) of the four treatment groups after 7.7 years, the loss rate of the group CLEAN was significantly different from all the other groups with surface preparation (PREP_FLOW + GROOVE + GROOVE_FLOW; *p* = 0.041)

Sixty‐four restorations (75.3%) out of 85 Class V restorations placed in 24 patients (10 male, 14 female) were available for follow‐up examination after an average period of 7.7 (± 0.35) years (shortest observation time: 6.8 years; longest observation time 8.2 years). The study investigated 33 restorations in male patients aged 70.9 (± 8.8) years and 31 in female patients aged 64.5 (± 11.9) years. Twenty‐eight restorations (43.75%) were located in maxillary teeth and 36 (56.25%) in mandibular teeth; Thirty restorations (46.88%) were placed in anterior teeth, 33 (51.56%) in premolars, and one (1.56%) in a molar. The distribution of examined restorations during the follow‐up was as follows: CLEAN: n = 12, PREP_FLOW: n = 12, GROOVE: n = 11 and GROOVE_FLOW: n = 18. A total of nine out of the 64 restorations available at the recall had been lost during the follow‐up period. Due to restoration replacement of unknown origin and subsequent treatment with a partial crown, two more restorations had to be excluded from evaluation. The total retention rate in the cohort investigated at 7.7 years, irrespective of the test groups, was 82.8%. Restorations placed without any dentin preparation (CLEAN group) showed the highest loss rate (27.8%); this difference was statistically significant when compared to the pooled groups which retrieved a pre‐treatment of the dentin surface (Group CLEAN vs. PREP_FLOW + GROOVE + GROOVE_FLOW; *p* = 0.041). There was no statistical difference between the treatment groups (Group PREP_FLOW vs. GROOVE vs. GROOVE_FLOW, *p* = 0.328. Figure 2 shows the loss rates of the treatment groups.

Based on modified USPHS criteria, the test groups did not differ significantly in esthetic appearance, marginal adaptation, anatomic form, and marginal discoloration (see Table 5).

**TABLE 5:** Examination results based on modified USPHS criteria

USPHS ratings	Group 1 CLEAN (n = 12)	Group 2 PREP_FLOW (n = 12)	Group 3 GROOVE (n = 11)	Group 4 GROOVE_FLOW (n = 18)
**Esthetic appearance**				
Alpha	91.7%	75%	100%	83.3%
Bravo	8.3%	25%	0%	16.7%
**Marginal adaptation**				
Alpha	75%	75%	81.8%	77.8%
Bravo	25%	25%	18.2%	22.2%
**Anatomic form**				
Alpha	100%	91.7%	90.9%	88.9%
Bravo	0%	8.3%	9.1%	11.1%
**Marginal discoloration**				
Alpha	83.3%	58.3%	81.8%	66.7%
Bravo	16.7%	41.7%	18.2%	27.8%
Charlie	0%	0%	0%	5.6%

All restorations in the group “GROOVE” examined at 7.7 years properly matched the shade of the adjacent tooth structure. In contrast, one‐quarter of the restorations in Group 2 (PREP_FLOW) had a slight to moderate, but still esthetically acceptable, mismatch in shade. Continuous transitions between restoration and tooth structure resulting in a natural anatomic form were found in all restorations of the “CLEAN” group. Approximately 10% of the restorations in the other groups were over‐ or under‐contoured. This difference was not statistically significant (*p* = 0.716). The restorations in Group 2 (PREP_FLOW) were most frequently (41.7%) affected by partial marginal discoloration. In Groups 1 and 3 (CLEAN and GROOVE), the prevalence of partial marginal discoloration was lower (16.7% and 18.2%). One restoration (5.6%) in Group 4 (GROOVE_FLOW) was rated Charlie for marginal discoloration. The differences between the groups described were not statistically significant (*p* = 0.613). The ratings of the restorations by the two investigators were in high agreement (ĸ = 1).

**FIGURE 3:**
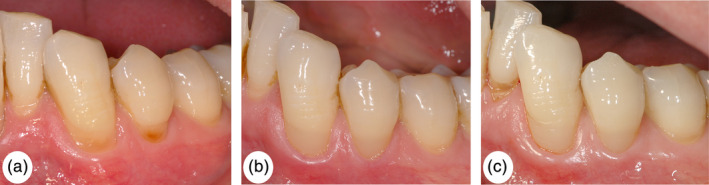
(a) Cervical defects on teeth 32, 33 and 34, pre‐operative situation, male patient aged 64. The defects on teeth 33 and 34 were included into the study. (b) Restorations 33 and 34 during the first postoperative examination after one week. (c) Restoration during follow‐up after >7 years. The restoration on tooth 33 was rated “Bravo” for marginal adaptation and marginal discoloration, and “Alpha” for all the other criteria. The restoration on tooth 34 was rated “Alpha” for all criteria

Figure 3a‐c shows a clinical case before treatment, one week after the treatment was performed and after the follow‐up period of >7 years.

Comparisons between the treatment groups with regard to bleeding on probing (BOP) did not show significant differences for any of the six probing sites (*p* = 0.323). One case in the CLEAN group was rated Bravo for gingival response (USPHS criteria: gingival response without clinical inflammation, see Table 4).

None of the teeth available at the recall showed secondary caries, and hypersensitivity was only reported in one case.

## 4 DISCUSSION

This clinical long‐term study investigated the performance of composite restorations placed in NCCLs depending on the cavity pre‐treatment. Restorations without any dentin preparation showed the highest loss rate at 7.7 years. Therefore, the null hypothesis has to be rejected in regard to the retention rate.

The challenges of clinical studies include increasing patient drop‐out rates and restoration losses (Peumans, De Munck, Van Landuyt, & Van Meerbeek, 2015; van Dijken & Lindberg, 2015). The patient cohort participating in this study was at an advanced age, which is common in investigations dealing with restorations placed in NCCLs (Kim, Cho, Lee, & Cho, 2017; van Dijken, 2010; van Dijken & Pallesen, 2012). At baseline, 54% of the patients were over 60 years of age. With a follow‐up period of 7.7 years and 75.3% of the restorations available at the recall, this investigation can be described as a long‐term study with a medium‐range recall rate, based on the classification by Peumans, De Munck, Mine, and Van Meerbeek (2014). The total retention rate of 82.8% observed at 7.7 years is comparable to values found in the literature (Mahn, Rousson, & Meta, 2015; van Dijken, 2010). In a meta‐analysis addressing the influence of bonding systems on the clinical long‐term stability of cervical restorations, retention rates were 82.6% at 5 years and 67.7% at 8 years (Mahn et al., 2015).

The clinical success of cervical restorations depends mainly on their adhesion to the tooth structure, due to the lack of mechanical retention. One‐step self‐etch adhesives show an inferior “clinical index” when compared to two‐step self‐etch or three‐step etch & rinse systems in this indication (Mahn et al., 2015). The “clinical index” summarizes in‐vivo success, taking into account the clinical results for retention loss, marginal discoloration and marginal adaptation (Heintze, Ruffieux, & Rousson, 2010). In our study, we placed adhesively bonded composite restorations. This approach was selected on the basis of favorable clinical data from other in‐vivo studies (Peumans et al., 2014; Peumans et al., 2015; van Dijken & Lindberg, 2015). In our study we used the adhesive Syntac Classic in a selective enamel etching mode (Ivoclar Vivadent, Schaan, Liechtenstein) in combination with the system‐inherent self‐etching primer. This three‐step adhesive (Syntac Classic) applied with selective enamel etching in NCCLs showed the lowest annual failure rate in a follow‐up period of 13 years and is considered to be a reliable unfilled system (Korner, Sulejmani, Wiedemeier, Attin, & Tauböck, 2018; van Dijken & Pallesen, 2008). Enamel etching is associated with improved marginal adaptation, but not crucial to the retention rates of restorations (Peumans, De Munck, Van Landuyt, Lambrechts, & Van Meerbeek, 2007; Ritter, Heymann, Swift Jr, Sturdevant, & Wilder Jr, 2008).

Dentin in cervical lesions shows highly sclerotic, hypermineralized surface structures with obliterated dentinal tubules (Eliguzeloglu Dalkilic & Omurlu, 2012; Sakoolnamarka, Burrow, Prawer, & Tyas, 2000; Tay & Pashley, 2004). These structures cannot be completely removed by etching and cause insufficient dentin hybridization when left in place (Sakoolnamarka et al., 2000; Tay & Pashley, 2004). Clinical investigations have shown additional roughening of the dentin surface to be associated with significantly lower failure rates at times (Eliguzeloglu Dalkilic & Omurlu, 2012; Mahn et al., 2015; van Dijken, 2010). The reason is that mechanical preparation removes sclerotic dentin, which prevents the formation of an adequate hybrid layer (Mahn et al., 2015; Van Meerbeek, Braem, Lambrechts, & Vanherle, 1994). This approach has not yet become sufficiently established in clinical practice, although it may be essential to the long‐term survival of Class V restorations (Mahn et al., 2015). The adhesive used in our study was originally designed for the “selective etch‐technique” and chosen because it was considered to be the “golden standard” at the starting point of our investigation in 2007. Nevertheless, both approaches, that is, self‐etch and etch & rinse, have a lower in‐vitro bond strength to sclerotic dentin, as compared to normal dentin (Karakaya et al., 2008; Kwong et al., 2002). Our results support these in‐vitro data, since our treatment group without any preparation, that is, without removal of sclerotic superficial dentin, showed the highest loss rate at 7.7 years.

A systematic review and a meta‐analysis addressed the question as to whether flowables improve the marginal adaptation, marginal discoloration and retention of NCCL restorations, when compared to conventional composites (Szesz et al., 2017). Flowables showed better marginal adaptation and similar marginal discoloration, although the level of evidence was questionable. Composite viscosity did not seem to influence retention rates at 3 years (Szesz et al., 2017). The use of a flowable composite as a layer between dentin and a conventional composite improved marginal adaptation in vitro (Li et al., 2006). Our study does not support this result, as there were no significant differences in marginal adaptation between the treatment groups (see Table 5). Generally, it should be noted that there are only limited long‐term data on adhesive restorations placed in non‐carious Class V cavities. Most studies used follow‐up periods of 3 years or less; only few investigators selected periods of 5 years or more (Peumans, Wouters, De Munck, Van Meerbeek, & Van Landuyt, 2018; van Dijken & Pallesen, 2012). Besides, the study designs are heterogeneous. A distinction should be made as to whether a flowable composite is only used to line the cervical cavity surface, as in our study, or to fill the entire cavity. When using flowables cervically (dentin/cementum) and conventional composites coronally (enamel), marginal adaptation and marginal discoloration should be rated separately for the two different margins. This is a limitation of our study; we only rated the restoration as a whole, without differentiating between coronal and cervical margins.

In addition, we investigated whether the preparation of a minimally invasive (i.e., 0.5 mm) groove influences Class V restorations. The main reason for this cavity design was to reduce thin layers of composite in epi‐/subgingival areas adjacent to the cavity margin. Without a precisely defined cavity margin, composite overhangs might interfere with clinical parameters such as gingival inflammation, marginal discoloration etc. Our results showed that the preparation of a groove in the cervical marginal area has no benefit on the clinical outcome in Class V cavities. This might be explained by our clinical treatment protocol: the retraction cord was placed in an atraumatic way (see materials and methods), which ensured a good overview over the treatment area. In groups were a flowable was used, the placing of the first viscous composite increment was eased, as the oxygen inhibition layer seemed to be thinner when compared to Heliobond, and the surface was therefore less “slippery.” Also, for finishing of the cervical restoration margins, oscillating files (EVA System (KaVo, Biberach, Germany) and Proxoshape PS2 (Intensiv, Montagnola, Switzerland)) were used, which ensured the removal of overhangs. However, this cavity design was not addressed by any scientific publications so far, which hampers a comparison with other clinical studies. Nevertheless, the cervical groove and the use of a flowable ease the manipulation of the composite, as a more sticky surface exists and a clear margin is visible.

Secondary caries was not found in any of the restorations examined. It was also a rare occurrence in other studies of restorations placed in NCCL restorations (Peumans et al., 2015). However, participants in clinical studies typically show good oral hygiene and a low caries risk. Moreover, only non‐carious cervical defects were restored (Nedeljkovic, Teughels, De Munck, Van Meerbeek, & Van Landuyt, 2015). This finding is in agreement with our results. The non‐significant differences in gingival response at the cervical restoration margin between the treatment groups may be explained by the fact that the cavity margins were epigingival or slightly supragingival at the recall (approx. 7.7 years postoperative) as a result of age‐related gingival recession.

## 5 CONCLUSION AND CLINICAL SIGNIFICANCE

Restorations placed without any dentin preparation showed the highest loss rate at 7.7 years. Roughening of the dentin surface, and/or the preparation of a fine groove led to a higher long‐term survival of restorations placed in NCCLs and can be included into the clinical treatment protocol of NCCLs.

## CONFLICT OF INTEREST

The authors do not have any financial interest in the companies whose materials are included in this article. The authors declare that they have nothing to disclose.

## References

[cre2310-bib-0001] Aw, T. C. , Lepe, X. , Johnson, G. H. , & Mancl, L. (2002). Characteristics of noncarious cervical lesions: A clinical investigation. The Journal of the American Dental Association, 133, 725–733. 12083648 10.14219/jada.archive.2002.0268

[cre2310-bib-0002] Borcic, J. , Anic, I. , Urek, M. M. , & Ferreri, S. (2004). The prevalence of non‐carious cervical lesions in permanent dentition. Journal of Oral Rehabilitation, 31, 117–123. 15009594 10.1046/j.0305-182x.2003.01223.x

[cre2310-bib-0003] Borges, A. L. , Borges, A. B. , Xavier, T. A. , Bottino, M. C. , & Platt, J. A. (2014). Impact of quantity of resin, C‐factor, and geometry on resin composite polymerization shrinkage stress in Class V restorations. Operative Dentistry, 39, 144–151. 23786611 10.2341/12-440-L

[cre2310-bib-0004] Boushell, L. W. , Heymann, H. O. , Ritter, A. V. , Sturdevant, J. R. , Swift, E. J., Jr. , Wilder, A. D., Jr. , … Walter, R. (2016). Six‐year clinical performance of etch‐and‐rinse and self‐etch adhesives. Dental Materials, 32, 1065–1072. 27352732 10.1016/j.dental.2016.06.003

[cre2310-bib-0005] Cieplik, F. , Scholz, K. J. , Tabenski, I. , May, S. , Hiller, K. A. , Schmalz, G. , … Federlin, M. (2017). Flowable composites for restoration of non‐carious cervical lesions: Results after five years. Dental Materials, 33, e428–e437. 29102158 10.1016/j.dental.2017.09.012

[cre2310-bib-0006] Correia, A. M. O. , Tribst, J. P. M. , Matos, F. S. , Platt, J. A. , Caneppele, T. M. , & Borges, A. L. (2018). Polymerization shrinkage stresses in different restorative techniques for non‐carious cervical lesions. Journal of Dentistry, 76, 68–74. 29935253 10.1016/j.jdent.2018.06.010

[cre2310-bib-0007] Craig, R. G. , & Peyton, F. A. (1958). Elastic and mechanical properties of human dentin. Journal of Dental Research, 37, 710–718. 13563732 10.1177/00220345580370041801

[cre2310-bib-0008] Cvar, J. F. , & Ryge, G. (2005). Reprint of criteria for the clinical evaluation of dental restorative materials. Clinical Oral Investigations, 9, 215–232. 16315023 10.1007/s00784-005-0018-z

[cre2310-bib-0009] Dickson, W. J. , Vandewalle, K. S. , Lien, W. , Dixon, S. A. , & Summitt, J. B. (2015). Effects of cyclic loading and toothbrush abrasion on cervical lesion formation. General Dentistry, 63, e1–e5. 25734292

[cre2310-bib-0010] El‐din, A. K. , Miller, B. H. , & Griggs, J. A. (2004). Resin bonding to sclerotic, noncarious, cervical lesions. Quintessence International, 35, 529–540. 15259968

[cre2310-bib-0011] Eliguzeloglu Dalkilic, E. , & Omurlu, H. (2012). Two‐year clinical evaluation of three adhesive systems in non‐carious cervical lesions. Journal of Applied Oral Science, 20, 192–199. 22666836 10.1590/S1678-77572012000200012 PMC3894762

[cre2310-bib-0012] Fennis, W. M. , Kuijs, R. H. , Barink, M. , Kreulen, C. M. , Verdonschot, N. , & Creugers, N. H. (2005). Can internal stresses explain the fracture resistance of cusp‐replacing composite restorations? European Journal of Oral Sciences, 113, 443–448. 16202034 10.1111/j.1600-0722.2005.00233.x

[cre2310-bib-0013] Hakimeh, S. , Vaidyanathan, J. , Houpt, M. L. , Vaidyanathan, T. K. , & von Hagen, S. (2000). Microleakage of compomer class V restorations: Effect of load cycling, thermal cycling, and cavity shape differences. The Journal of Prosthetic Dentistry, 83, 194–203. 10668032 10.1016/s0022-3913(00)80012-8

[cre2310-bib-0014] Heintze, S. D. , Ruffieux, C. , & Rousson, V. (2010). Clinical performance of cervical restorations—a meta‐analysis. Dental Materials, 26, 993–1000. 20638116 10.1016/j.dental.2010.06.003

[cre2310-bib-0015] Igarashi, Y. , Yoshida, S. , & Kanazawa, E. (2017). The prevalence and morphological types of non‐carious cervical lesions (NCCL) in a contemporary sample of people. Odontology, 105, 443–452. 28275876 10.1007/s10266-017-0300-y

[cre2310-bib-0016] Karakaya, S. , Unlu, N. , Say, E. C. , Oezer, F. , Soyman, M. , & Tagami, J. (2008). Bond strengths of three different dentin adhesive systems to sclerotic dentin. Dental Materials Journal, 27, 471–479. 18717178 10.4012/dmj.27.471

[cre2310-bib-0017] Karaman, E. , Yazici, A. R. , Ozgunaltay, G. , & Dayangac, B. (2012). Clinical evaluation of a nanohybrid and a flowable resin composite in non‐carious cervical lesions: 24‐month results. The Journal of Adhesive Dentistry, 14, 485–492. 22724113 10.3290/j.jad.a27794

[cre2310-bib-0018] Karan, K. , Yao, X. , Xu, C. , & Wang, Y. (2009). Chemical profile of the dentin substrate in non‐carious cervical lesions. Dental Materials, 25, 1205–1212. 19464050 10.1016/j.dental.2009.04.006 PMC2731826

[cre2310-bib-0019] Kim, J. H. , Cho, J. , Lee, Y. , & Cho, B. H. (2017). The survival of Class V composite restorations and analysis of marginal discoloration. Operative Dentistry, 42, E93–E101. 28467254 10.2341/16-186-C

[cre2310-bib-0020] Kolak, V. , Pesic, D. , Melih, I. , Lalović, M. , Nikitović, A. , & Jakovljević, A. (2018). Epidemiological investigation of non‐carious cervical lesions and possible etiological factors. Journal of Clinical and Experimental Dentistry, 10, e648–e656. 30057705 10.4317/jced.54860 PMC6057075

[cre2310-bib-0021] Korner, P. , Sulejmani, A. , Wiedemeier, D. B. , Attin, T. , & Tauböck, T. T. (2018). Demineralized enamel reduces margin integrity of self‐etch, but not of etch‐and‐rinse bonded composite restorations. Odontology, 107, 308–315. 30467810 10.1007/s10266-018-0398-6

[cre2310-bib-0022] Kwong, S. M. , Cheung, G. S. , Kei, L. H. , Itthagarun, A. , Smales, R. J. , Tay, F. R. , & Pashley, D. H. (2002). Micro‐tensile bond strengths to sclerotic dentin using a self‐etching and a total‐etching technique. Dental Materials, 18, 359–369. 12175574 10.1016/s0109-5641(01)00051-3

[cre2310-bib-0023] Li, Q. , Jepsen, S. , Albers, H. K. , & Eberhard, J. (2006). Flowable materials as an intermediate layer could improve the marginal and internal adaptation of composite restorations in Class‐V‐cavities. Dental Materials, 22, 250–257. 16084584 10.1016/j.dental.2005.04.011

[cre2310-bib-0024] Mahn, E. , Rousson, V. , & Meta, H. S. (2015). Analysis of the influence of bonding parameters on the clinical outcome of tooth‐colored cervical restorations. The Journal of Adhesive Dentistry, 17, 391–403. 26525003 10.3290/j.jad.a35008

[cre2310-bib-0025] Mullejans, R. , Lang, H. , Schuler, N. , Baldawi, M. O. , & Raab, W. H. (2003). Increment technique for extended class V restorations: An experimental study. Operative Dentistry, 28, 352–356. 12877419

[cre2310-bib-0026] Nedeljkovic, I. , Teughels, W. , De Munck, J. , Van Meerbeek, B. , & Van Landuyt, K. L. (2015). Is secondary caries with composites a material‐based problem? Dental Materials, 31, e247–e277. 26410151 10.1016/j.dental.2015.09.001

[cre2310-bib-0027] Osborne‐Smith, K. L. , Burke, F. J. , & Wilson, N. H. (1999). The aetiology of the non‐carious cervical lesion. International Dental Journal, 49, 139–143. 10858746 10.1002/j.1875-595x.1999.tb00898.x

[cre2310-bib-0028] Palamara, J. E. , Palamara, D. , Messer, H. H. , & Tyas, M. J. (2006). Tooth morphology and characteristics of non‐carious cervical lesions. Journal of Dentistry, 34, 185–194. 16112334 10.1016/j.jdent.2005.05.005

[cre2310-bib-0029] Pecie, R. , Krejci, I. , Garcia‐Godoy, F. , & Bortolotto, T. (2011). Noncarious cervical lesions‐‐a clinical concept based on the literature review. Part 1: Prevention. American Journal of Dentistry, 24, 49–56. 21469407

[cre2310-bib-0030] Perez, C. R. (2010). Alternative technique for class V resin composite restorations with minimum finishing/polishing procedures. Operative Dentistry, 35, 375–379. 20533640 10.2341/09-310-TR

[cre2310-bib-0031] Perez, C. R. , Gonzalez, M. R. , Prado, N. A. , De Miranda, M. S. , Macêdo, M. D. , & Fernandes, B. M. (2012). Restoration of noncarious cervical lesions: When, why, and how. International Journal of Dentistry, 2012, 687058. 22216032 10.1155/2012/687058 PMC3246729

[cre2310-bib-0032] Peumans, M. , De Munck, J. , Mine, A. , & Van Meerbeek, B. (2014). Clinical effectiveness of contemporary adhesives for the restoration of non‐carious cervical lesions. A systematic review. Dental Materials, 30, 1089–1103. 25091726 10.1016/j.dental.2014.07.007

[cre2310-bib-0033] Peumans, M. , De Munck, J. , Van Landuyt, K. , Lambrechts, P. , & Van Meerbeek, B. (2007). Five‐year clinical effectiveness of a two‐step self‐etching adhesive. The Journal of Adhesive Dentistry, 9, 7–10. 17432395

[cre2310-bib-0034] Peumans, M. , De Munck, J. , Van Landuyt, K. , & Van Meerbeek, B. (2015). Thirteen‐year randomized controlled clinical trial of a two‐step self‐etch adhesive in non‐carious cervical lesions. Dental Materials, 31, 308–314. 25637318 10.1016/j.dental.2015.01.005

[cre2310-bib-0035] Peumans, M. , Wouters, L. , De Munck, J. , Van Meerbeek, B. , & Van Landuyt, K. (2018). Nine‐year clinical performance of a HEMA‐free one‐step self‐etch adhesive in noncarious cervical lesions. The Journal of Adhesive Dentistry, 20, 195–203. 29904752 10.3290/j.jad.a40630

[cre2310-bib-0036] Ritter, A. V. , Heymann, H. O. , Swift, E. J., Jr. , Sturdevant, J. R. , & Wilder, A. D., Jr. (2008). Clinical evaluation of an all‐in‐one adhesive in non‐carious cervical lesions with different degrees of dentin sclerosis. Oper Dent., 33, 370–378. 18666493 10.2341/07-128

[cre2310-bib-0037] Sadaf, D. , & Ahmad, Z. (2014). Role of brushing and occlusal forces in non‐carious cervical lesions (NCCL). International Journal of Biomedical Sciences, 10, 265–268. PMC4289701 25598758

[cre2310-bib-0038] Sakoolnamarka, R. , Burrow, M. F. , Prawer, S. , & Tyas, M. J. (2000). Micromorphological investigation of noncarious cervical lesions treated with demineralizing agents. The Journal of Adhesive Dentistry, 2, 279–287. 11317374

[cre2310-bib-0039] Smith, B. G. , & Knight, J. K. (1984). An index for measuring the wear of teeth. British Dental Journal, 156, 435–438. 6590081 10.1038/sj.bdj.4805394

[cre2310-bib-0040] Szesz, A. , Parreiras, S. , Martini, E. , Reis, A. , & Loguercio, A. (2017). Effect of flowable composites on the clinical performance of non‐carious cervical lesions: A systematic review and meta‐analysis. Journal of Dentistry, 65, 11–21. 28729119 10.1016/j.jdent.2017.07.007

[cre2310-bib-0041] Tay, F. R. , & Pashley, D. H. (2004). Resin bonding to cervical sclerotic dentin: A review. Journal of Dentistry, 32, 173–196. 15001284 10.1016/j.jdent.2003.10.009

[cre2310-bib-0042] van Dijken, J. W. (2010). A prospective 8‐year evaluation of a mild two‐step self‐etching adhesive and a heavily filled two‐step etch‐and‐rinse system in non‐carious cervical lesions. Dental Materials, 26, 940–946. 20646753 10.1016/j.dental.2010.05.009

[cre2310-bib-0043] van Dijken, J. W. , & Lindberg, A. (2015). A 15‐year randomized controlled study of a reduced shrinkage stress resin composite. Dental Materials, 31, 1150–1158. 26205382 10.1016/j.dental.2015.06.012

[cre2310-bib-0044] van Dijken, J. W. , & Pallesen, U. (2008). Long‐term dentin retention of etch‐and‐rinse and self‐etch adhesives and a resin‐modified glass ionomer cement in non‐carious cervical lesions. Dental Materials, 24, 915–922. 18155288 10.1016/j.dental.2007.11.008

[cre2310-bib-0045] van Dijken, J. W. , & Pallesen, U. (2012). A 7‐year randomized prospective study of a one‐step self‐etching adhesive in non‐carious cervical lesions. The effect of curing modes and restorative material. Journal of Dentistry, 40, 1060–1067. 22955004 10.1016/j.jdent.2012.08.017

[cre2310-bib-0046] Van Meerbeek, B. , Braem, M. , Lambrechts, P. , & Vanherle, G. (1994). Morphological characterization of the interface between resin and sclerotic dentine. Journal of Dentistry, 22, 141–146. 8027456 10.1016/0300-5712(94)90197-x

[cre2310-bib-0047] Walter, C. , Kress, E. , Gotz, H. , Taylor, K. , Willershausen, I. , & Zampelis, A. (2014). The anatomy of non‐carious cervical lesions. Clinical Oral Investigations, 18, 139–146. 23494453 10.1007/s00784-013-0960-0

[cre2310-bib-0048] Wiegand, A. , & Schlueter, N. (2014). The role of oral hygiene: Does toothbrushing harm? Monographs in Oral Science, 25, 215–219. 24993269 10.1159/000360379

[cre2310-bib-0049] Yang, J. , Cai, D. , Wang, F. , He, D. , Ma, L. , Jin, Y. , & Que, K. (2016). Non‐carious cervical lesions (NCCLs) in a random sampling community population and the association of NCCLs with occlusive wear. Journal of Oral Rehabilitation, 43, 960–966. 27658541 10.1111/joor.12445

